# Evaluation of Hermetic Storage Bags for the Preservation of Yellow Maize in Poultry Farms in Dormaa Ahenkro, Ghana

**DOI:** 10.3390/insects14020141

**Published:** 2023-01-31

**Authors:** Bismark Opoku, Enoch Adjei Osekre, George Opit, Augustine Bosomtwe, Georgina V. Bingham

**Affiliations:** 1 Department of Crop and Soil Sciences, Kwame Nkrumah University of Science and Technology, Private Mail Bag, Kumasi, Ghana; 2 Department of Entomology and Plant Pathology, Oklahoma State University, 127 Noble Research Center, Stillwater, OK 74078, USA; 3 CSIR-Plant Genetic Resources Research Institute, Bunso P.O. Box 7, Ghana; 4 Department of Entomology, University of Nebraska-Lincoln, 103 Entomology Hall, Lincoln, NE 68583, USA

**Keywords:** airtight storage bag, stored products insect, mycotoxin, yellow food grain

## Abstract

**Simple Summary:**

The effectiveness of the ZeroFly^®^ Hermetic and Purdue Improved Crop Storage bags to keep insect pest and mycotoxin levels in check in yellow maize was compared to that of standard polypropylene bag storage at three poultry farms in Dormaa Ahenkro, Ghana. This study provides data on insect numbers, insect-damaged kernels, weight loss of maize, mycotoxin (aflatoxin and fumonisin) levels and proximate composition of maize in the three types of storage bags after six months of storage. Data showed that ZeroFly^®^ Hermetic and Purdue Improved Crop Storage bags protected yellow maize against insect pests and held aflatoxin and fumonisin levels within recommended thresholds.

**Abstract:**

Using low-quality maize, resulting from insect pests and fungal attack, for formulating feed reduces chicken performance. This study evaluated the effectiveness of hermetic storage bags to keep insect pest and mycotoxin levels in check in yellow maize. The study was conducted in storehouses at three poultry farms in Dormaa Ahenkro, Bono Region, Ghana. The experiment was set up in a Randomized Complete Block Design with ZeroFly® Hermetic (ZFH), Purdue Improved Crop Storage (PICS), and Polypropylene (PP) bags as treatments. In each treatment, twelve 50 kg samples of untreated maize were each put in 100 kg capacity bags. Two bags in each treatment were destructively sampled monthly for 6 months. The number of insects was significantly higher in the PP bag (161.00 ± 4.25), compared to the PICS and ZFH bags: 7.00 ± 0.29 and 4.50 ± 0.76, respectively. The PICS and ZFH bags had less insect damage and lower weight loss than the PP bags. Aflatoxin and fumonisin levels were below the recommended safe thresholds of 15 ppb and 4 ppm, respectively, in all the bags. With the exception of ash, proximate analyses were higher for all variables in the PICS and ZFH bags. The study showed that PICS and ZFH bags conserved maize quality better than the PP bag.

## 1. Introduction

Maize (*Zea mays* L.) is one of the most important cereals widely cultivated throughout the world. It is a major staple food in Africa, and contributes considerably to the food and income security of many smallholder farmers [1,2]. According to [3] maize production in Africa was around 75 million tons in 2018, representing 7.5% of global maize production. In Ghana, maize ranks first in terms of area planted, and accounts for 50–60% of total cereal production [4]. In spite of its economic and food security importance, maize producers in developing countries experience significant post-harvest losses due to poor storage [5,6]. Global grain production of wheat, maize, rice, and soybean crops peaked at a little above 2 million tons (mt) of harvested grain in 2018. Pre-harvest losses due to biotic and abiotic stresses are estimated at about 35% of the total possible biological product of 3153 mt, with 1051.5 mt being lost before harvest [7]. In most African countries, annual loss of stored maize due to insect infestation alone is estimated to be 20–50% [8].

Delaying harvest and heaping of harvested cobs (ears) on the ground in the field in an attempt to dry the ears to safe moisture content (MC) levels before storage are common practices among smallholder farmers in Ghana [9], but such practices expose the grain to increased pest and fungal attack, resulting in greater post-harvest losses. On the other hand, demand for maize in Ghana is rising as a result of its use in poultry feed in the burgeoning poultry industry. Most of the demand is for yellow maize because poultry farmers prefer it for egg production. The current maize deficit in Ghana is addressed through importation. Ref. [10] reported that when yellow maize is used to feed poultry, it gives a deep yellow coloration to egg yolk, poultry skin, and animal fats which consumers attribute to healthiness and freshness.

Insects, rodents and fungi are known to cause considerable damage to stored grains [8]. Insect infestation causes direct loss through feeding, and indirectly, by increasing moisture content which predisposes the grain to infection by mycotoxin-producing fungi such as *Aspergillus flavus* [1]. In Ghana the most abundant *Sitophilus* species on stored maize is *Sitophilus zeamais* Mots. (Coleoptera: Curculionidae). Mycotoxins including aflatoxin and fumonisin are poisonous secondary metabolites which contaminate durable food commodities such as maize and groundnuts. They have been found to be carcinogenic, and have been linked to growth retardation, neurological impairment, immunosuppression, and mortality in humans and animals [11]. Estimates indicate that approximately 25% of the world’s food crops are contaminated with mycotoxins, but in sub-Saharan Africa (SSA), contamination is much greater [12].

Without proper drying and storage, insect infestation and mycotoxin concentrations increase rapidly in the warm, humid environment found in tropical regions, making post-harvest losses pervasive [13,14]. Due to inadequate or lack of effective storage technologies, smallholder farmers in developing countries typically sell or use their produce immediately after harvest to evade losses [15,16]. If grains are stored, jute and polypropylene bags are the popular storage methods in many African countries [17,18]; however, these traditional bags do not adequately preserve stored commodities.

Storage of grains in hermetic bags to minimize losses is being promoted in Africa [2,17]. In Ghana several studies [19,20,21,22] on hermetic storage bags for the protection of commodities against stored product pests have focused on the Purdue Improved Crop Storage (PICS) bag, which is a triple bagging hermetic technology consisting of two liners and an outer woven layer of polypropylene. However, a recent study by [23] found that the deltamethrin-incorporated polypropylene hermetic storage bag (ZeroFly^®^ Hermetic storage bag) produced by Vestergaard SA, Lausanne, Switzerland, also offers effective protection to stored maize against insect pests and mycotoxins. Unlike the PICS bag, the ZeroFly^®^ Hermetic storage bag (hereafter referred to as ZFH bag) comprises a single inner liner with an outer deltamethrin-incorporated polypropylene fabric.

Studies on the ZFH bag to date have been conducted using white maize, and under laboratory conditions, i.e., not in the field. However, poultry farmers in Ghana use yellow maize as the main component in their poultry feed, and typically store the maize in polypropylene (PP) bags which provide little to no preservation. In PP bags, insect pest infestation and mycotoxin contamination usually reduce maize quality, and consequently lower feed quality, hence negatively affecting chicken performance. It is also instructive to note that the use of jute bags by farmers for maize storage is being discontinued in Ghana and the jute bags replaced with PP and hermetic storage bags. Using ZFH bags likely becomes attractive to poultry farmers if the perceived benefits are shown to substantially outweigh the cost.

Therefore, in this study, we evaluated the effectiveness of the ZFH bag to protect yellow maize against insect infestation and mycotoxin contamination in poultry farms in Ghana.

## 2. Materials and Methods

### 2.1. Study Site and Set up of Treatments

The study was conducted in the storehouses of three poultry farms; Evans Joes Farms, M. M. Unity Farms, and T. K. Takyi Farms, all in Dormaa Ahenkro, Ghana (7°17′ N 2°53′ W). Three types of storage bags served as the experimental treatments, and these were the ZeroFly^®^ Hermetic Storage bag (ZFH), Purdue Improved Crop Storage bag (PICS), and PP bag. The ZeroFly^®^ storage bags were obtained from Vestergaard’s distributor in Nigeria (Turner Wright Nigeria Limited 15, Adenekan Salako Close, Ogba, Lagos, Nigeria), whereas the PICS and PP bags were obtained from a local distributor in Kumasi (Bentronic Productions, Kumasi, Ghana). The study set-up was a Randomized Complete Block Design (RCBD) with three treatments. Each treatment was replicated three times with each poultry farm storehouse being a replicate or block, and there was a sub-replication of two, where two bags in each treatment in each farm were destructively sampled monthly. One hundred and eight 50 kg bags of clean untreated (insecticide-free) yellow maize (Abontem variety) of ~13% MC were purchased directly from a farmer named Aduamere Owusu from Sunyani-Chiraa, Ghana in 2022 and transported to a single storehouse location in bags provided by the producer. The maize was emptied onto a tarpaulin and thoroughly mixed, and 50 kg was transferred to each of the 100 kg capacity ZFH, PICS, and PP bags used for the study. In each storehouse, there were twelve 100 kg capacity ZFH, PICS and PP bags, each containing 50 kg of yellow maize, that is, altogether thirty-six 100 kg capacity bags in each storehouse. Thus, a total of 108 bags were used for the three poultry houses; twelve bags of each type in a house. The twelve bags for each treatment were arranged on two wooden pallets, that is, six bags on each of the pallets. Wooden pallets were used to prevent maize in the bags from absorbing moisture from the floor. The pallets for each treatment were separated by 2 m of free space. The integrity of the inner liners of the hermetic bags was checked before use to ensure there were no punctures, and were then checked again to ensure they were tightly sealed after the maize was put in, to maintain hermeticity. The outer polypropylene layers were equally checked to ensure they were not torn before putting the maize into the bags.

### 2.2. Sampling and Data Collection

Initial sampling was done at the start of the study; samples were subsequently taken monthly for six months. During monthly sampling, bags on each pallet were numbered 1 to 6, and pieces of paper were also numbered similarly. The pieces of paper were folded and placed in a container, mixed up, and two were randomly picked to determine which bags in each treatment were selected for sampling and data collection. Therefore, there was a sub-replication of two. Bags in the various treatments were destructively sampled each month. The bags of maize in each treatment, in the three storehouses, that were sampled each month were discontinued from the study.

#### 2.2.1. Determination of Moisture Content (MC)

The USAID Feed the Future Innovation Lab for the Reduction of Post-Harvest Loss moisture meter (hereafter referred to as PHL moisture meter, GrainMate) was used to determine the moisture content of maize in each sampling bag [24]. Each bag of maize that was chosen for data collection had the meter placed inside it. After five minutes of stabilizing, the meter’s display showed the grain equilibrium MC (EMC) (% wb), equilibrium relative humidity (% ERH), and temperature (°C). These values were then recorded. Three different readings were taken from each bag, and the mean MC for each bag was computed.

#### 2.2.2. Grain Sampling

Using a 1.2 m open-ended grain probe (Seedburo Equipment, Chicago, IL, USA), three 500 g samples of maize were obtained from each bag, one from the center and two from the sides. To guarantee homogeneity, the three samples from each bag were carefully blended in a 5 L plastic container and a 500 g sub-sample was weighed using a dial spring weighing scale (SP, CAMRY, Yongkang, PRC) and put in a plastic bag (39 cm × 25 cm) with labels. Later, these 500 g samples were used to determine kernel damage after sieving insects. Mycotoxin testing was performed on a second 500 g sample. To prevent additional fungal growth and development, maize samples for the mycotoxin testing were stored in a 17 L Koolatron 12 V Compact Portable Electric Cooler at −4 °C (P75, Koolatron Canada, Brantford, ON, Canada) and transported to the lab for processing.

#### 2.2.3. Estimation of Percentage Insect Damaged Kernels on Number Basis (%IDK)

In order to identify kernels with holes caused by insects, each 500 g sample of maize (on average there are 2689 ± 12.7 (SE) kernels in 500 g of yellow maize) collected for kernel damage evaluation was put onto a tray and all of the kernels were examined using a hand lens (Supertek Co. Ltd., Zhuhai city, China)(10×). To facilitate %IDK calculation, 100 g portions of the 2689 kernels were examined at a time until all the kernels were examined. Undamaged kernels and those with holes were separated, and the number of kernels in each category was counted. An electronic balance was used to measure the weight of insect-damaged kernels (IDK) (Mettler Toledo, LLC, Columbus, OH, USA) Batch No. PB302). The formula below was used to compute percent IDK (%IDK):

$$\mathrm{Percent}\;\mathrm{IDK}\;\left(\mathrm{number}\;\mathrm{basis}\right)=\frac{\mathrm{Number}\;\mathrm{of}\;\mathrm{IDK}}{\mathrm{Total}\;\mathrm{number}\;\mathrm{of}\;\mathrm{kernels}}\times 100$$

#### 2.2.4. Weight Loss

Percentage weight loss as a result of insect damage was determined using the count and weigh method of [25].

$$\mathrm{Weight}\;\mathrm{loss}\;(\%)=\frac{\left[\left(\mathrm{Wu}\;\times \;\mathrm{Nd}\right)-\left(\mathrm{Wd}\;\times \;\mathrm{Nu}\right)\right]}{\mathrm{Wu}\;\times \left(\mathrm{Nd}+\mathrm{Nu}\right)}\times 100$$

where Wu = Weight of undamaged grain, Nu = Number of undamaged grains, Wd = Weight of damaged grain, and Nd = Number of damaged grains.

#### 2.2.5. Extraction of Insects from Samples

U.S. Standard sieve #10 (2 mm openings) and #25 (0.71-mm openings) sieves (Dual Manufacturing Co., Franklin Park, IL, USA) were used to sift the 500 g maize samples that were collected to estimate the levels of insect infestation in order to recover insects. Using [26], insect species were identified, and the numbers of each species were recorded.

#### 2.2.6. Mycotoxin Analysis

The 500 g maize samples used were those specifically obtained for mycotoxin analysis. Aflatoxin and fumonisin analyses were conducted using AgraStrip^®^ Total Aflatoxin and Fumonisin quantitative test kits provided by Romer Labs^®^, Inc., Union, MO, USA. The AgraStrip^®^ Total Aflatoxin Quantitative Test is a one-step lateral flow immunochromatographic assay that determines a quantitative level for the presence of total aflatoxin. The AgraStrip^®^ Total Fumonisin Quantitative Test is a one-step lateral flow immunochromatographic assay that determines a quantitative level for the presence of total fumonisin. In both tests, sample grinding, extraction, solute preparation and test procedures were undertaken in accordance with the manufacturer’s instructions (Romer Labs Methods, romerlabs.com (Accessed on 10 February 2020).

#### 2.2.7. Determination of Nutrient Quality of Maize

Five hundred gram maize samples were taken from the various bags at the start and end of the six-month storage period, for proximate analysis, which was performed using the methods outlined in [27].

### 2.3. Data Analyses

The experimental design was a randomized complete block design (RCBD) with sub-replication. Statistical analyses were performed with SAS Version 9.4 (SAS Institute, Cary, NC, USA). The effects of sampling month (Month) and type of storage bag (Bag) were assessed using analysis of variance (ANOVA) methods with the poultry farm as the blocking factor (PROC MIXED). Analysis of the numbers of live insects was conducted with the use of a square root transformation, but untransformed values are reported. The simple effects of type of storage bag in a given month were assessed with protected planned contrasts (SLICE option in an LSMEANS statement). Additionally, the SLICE option was used to assess the simple effects of month in a given type of storage bag. Data analyses for response variables expressed as percentages were conducted with the use of an arcsine square root transformation to stabilize variances, but untransformed percentages are reported.

## 3. Results

### 3.1. Moisture Content (% MC) of Maize Sampled from Polypropylene (PP), Purdue Improved Crop Storage (PICS), and ZeroFly^®^ Hermetic (ZFH) Bags

The moisture content levels in maize from PP bags were significantly higher (*p* < 0.05) compared to the levels in PICS and ZFH bags during the 6 month storage period. Except for the second month, there were no significant differences observed in MC levels between PICS and ZFH bags in all the six months (Table 1). The MC level of maize in the PP bags increased from an initial 12.47 ± 0.03% to 15.51 ± 0.19% in month six. There were no differences in the MC of maize in ZFH bags throughout the storage period. Both PICS and ZFH bags maintained MC below 13%, which is the recommended MC for long-term storage of maize.

### 3.2. Number of Insects Sampled from Maize in Polypropylene (PP), Purdue Improved Crop Storage (PICS), and ZeroFly^®^ Hermetic (ZFH) Bags

*Sitophilus* species (adults) (Coleoptera: Curculionidae) were the only insects found in the initial maize samples. A negligible number of *Sitotroga cerealella* (Lepidoptera: Gelechiidae) was found in the PP bag samples only during the fourth month of sampling. By the second month, insects found in the maize samples from the hermetic bags were all dead. In contrast, insects found in the PP bag samples throughout the study were predominantly alive. There were no significant differences (*p* > 0.05) in the initial numbers of insects in the PP (0.83 ± 0.17), PICS (1.33 ± 0.44) and ZFH (1.17 ± 0.33) storage bags’ samples. The number of insects in the PP bag samples was consistently higher throughout the sampling period compared to the PICS and ZFH bags’ samples, reaching the highest number of 284.83 ± 25.58 in month four (Table 2). The highest number of insects sampled from maize in PICS (13.67 ± 4.00) and ZFH (12.17 ± 4.60) was after two months of storage, which was significantly higher than the initial number. However, insect numbers in the PICS and ZFH bags’ samples consistently declined from the third month onwards to 7.00 ± 0.29 and 4.50 ± 0.76, respectively, in month six.

### 3.3. Insect-Damaged Kernels (IDK) per 500 g of Maize Sampled from Polypropylene (PP), Purdue Improved Crop Storage (PICS), and ZeroFly^®^ Hermetic (ZFH) Storage Bags

The number of insect-damaged kernels (IDK) was significantly higher (*p* < 0.05) in PP bags compared to the two types of hermetic bags throughout the sampling periods. There were also significant differences in IDK within a storage bag for the different sampling months. However, there were no significant differences observed between the PICS and ZFH storage bags for the 6 month storage period (Table 3). From an initial mean of 0.83 ± 0.83, IDK in the PP increased significantly to its highest number (76.67 ± 0.44) after five months of storage. IDK in PP bags in the last three months were significantly higher than the first three months. For the hermetic storage bags, IDK was highest in PICS bags (12.00 ± 2.29) two months after storage, and in ZFH (9.50 ± 1.53) after one month of storage. Afterwards, there was a consistent and significant decline in IDK found in both types of hermetic bags, to their lowest numbers of 5.17 ± 0.44 and 2.83 ± 0.33 in PICS and ZFH, respectively.

### 3.4. Weight Loss (%) of Maize from Polypropylene (PP), Purdue Improved Crop Storage (PICS), and ZeroFly^®^ Hermetic (ZFH) Storage Bags

After one month of storage, a mean weight loss of 3.66 ± 1.15% was recorded in the PP bag which was not significantly different from the mean values of 1.05 ± 0.42% and 1.15 ± 0.58% for PICS and ZFH bags, respectively. From months two to six, significantly higher weight loss of maize was observed in the PP bag compared to the two hermetic bags, reaching the highest level (29.7 ± 7.07%) in month six. The weight loss recorded in PP bags in month six was significantly different from the rest of the sampling months. For PICS and ZFH bags, there were no significant differences in maize weight loss within all the sampling months (Table 4).

### 3.5. Levels of Aflatoxin in Maize Sampled from Polypropylene (PP), Purdue Improved Crop Storage (PICS), and ZeroFly^®^ Hermetic (ZFH) Bags

After one month of storage, aflatoxin levels were consistently higher in PP bags than in the PICS and ZFH bags throughout the following five months, reaching the highest level of 14.78 ± 0.12 ppb in month six (Table 5). There were no significant differences in aflatoxin levels between PICS and ZFH bags during all the sampling months. The highest mean total aflatoxin levels were 14.78 ± 0.12, 10.52 ± 0.14, and 10.38 ± 0.30 ppb in PP, PICS, and ZFH, respectively, at the end of the 6 month storage; these levels were all below the recommended safe threshold.

### 3.6. Levels of Fumonisin in Maize Sampled from Polypropylene (PP), Purdue Improved Crop Storage (PICS), and ZeroFly^®^ Hermetic (ZFH) Bags

Fumonisin levels in maize in PP bags were significantly higher compared to those in PICS and ZFH bags during the 6 month storage period. However, in all the three types of bag, the levels found were below the recommended safe threshold of 4 ppm (Table 6). No significant differences were recorded in the mean fumonisin levels in maize in PICS and ZFH bags within the sampling months.

### 3.7. Percentage of Crude Protein (%CPRO), Crude Fat (%CFAT), Carbohydrate (%CHO), Crude Fiber (%CFIBER), Ash Content (%ASH), Moisture Content (%MC) of Maize Sampled from Polypropylene (PP), Purdue Improved Crop Storage (PICS), and ZeroFly^®^ Hermetic (ZFH) Bags

There were no significant differences (*p* > 0.05) between the PICS and ZFH bags for the initial and final proximate analyses for all the variables. However, significant reduction of all the variables except %ASH was observed in maize in the PP bags compared to the two types of hermetic bags (Table 7). However, there was a significant increase in the percentage moisture content and percentage fiber from 12.29 ± 0.05 to 14.69 ± 0.50, and 2.37 ± 0.01 to 3.76 ± 0.61, respectively, in the PP bags at the end of the storage period.

## 4. Discussion

Moisture content of maize at the start of the storage period ranged between 12.47 and 12.62%, which was below the level (≤13%) recommended for safe storage of maize [28]. Even though the moisture content of maize was similar initially, it began to increase in the PP bags and remained significantly higher compared to levels in the PICS and ZFH bags during the 6 month storage period. The increase in MC could be attributed to the combined effect of entry of the outside humidity into the PP bags due to the porous nature of the PP bags and increasing numbers of live insects resulting in high respiration rate. According to [29], respiration by insects, molds and grains results in production of heat which enhances water vapor production to increase the MC of grains. A similar observation was made by [21] that MC increased in polypropylene and jute bags after storage of maize for 4 weeks. Moisture content in the hermetic bags was fairly consistent from the first to the sixth month. This could be due to lack of moisture exchange between the ambient environment and the grains in the inner liner of the hermetic bags. Another reason for the fairly consistent MC is the minimal insect activity in the hermetic bags which would otherwise increase grain temperature and moisture as a result of respiration [29]. This result is consistent with that of the study by [19] who worked on cocoa beans stored in hermetic cocoons.

The insect species found in sampled maize were *Sitophilus* spp. and *S*. *cerealella*, with the number of the latter being negligible. Greater numbers of live adult insects were recorded in maize in the PP bag than the hermetic bags throughout the sampling period. For the first two months, it appears insects were still able to utilize oxygen in the hermetic bags to multiply, however, after this period no live insects were found. Ref. [30] noted that the hermetic condition causes oxygen depletion and upsurge of carbon dioxide which affects the feeding, reproduction and survival of insects and microorganisms. The results of this study demonstrate that PICS and ZFH storage bags offer better protection to stored maize against insect pests than PP bags, by killing insects through oxygen depletion. Ref. [31] reported that when insects are enclosed in oxygen-limiting conditions, they die owing to desiccation because they are unable to generate the water needed to maintain vital life processes. For the ZFH bags, the deltamethrin incorporated in the yarns of the outer layer also served as a barrier to infestation from outside, giving maize an additional protection compared to PICS. This was evident when a lot of insects were found dead on the outer layer of the ZFH storage bags during sampling. This observation also corroborates the results of [23] in which most insects were found dead in ZeroFly^®^ Hermetic bags after six months of storage. High insect infestation in PP bags, which is the most common method of storing maize in Ghana, has been reported in numerous studies [32,33].

Grain damage by insects includes scarification of the pericarp, eating of the germ, and partial or complete consumption (hollowing) of the kernels [34]. This study showed that the number of insect-damaged kernels (IDK) was significantly higher in PP bags compared to the two types of hermetic bags throughout the sampling periods. The growth of IDK in the PP bags was progressive, as numbers increased with storage period, whereas the increase in IDK was kept in check in the hermetic storage bags. The higher number of IDK in the PP bags was due to feeding by the high numbers of live insects (*Sitophilus* spp. and *S. cerealella*) found in the PP bags. Both *Sitophilus* spp. and *S. cerealella* are internal feeders, and according to [35], internal feeders are more destructive because their larvae feed inside infested grains, and when they exit grains as adults, they leave highly visible exit holes which ultimately increases the number of IDK. The low number of IDK observed in the hermetic bags supports data from other studies on the storage of cowpea in hermetic bags [36,37,38]. One of the critical parameters considered by the Ghana Standards Authority (GSA) in grading maize is the percentage of insect-damaged kernels (%IDK). The acceptable %IDK threshold by wholesalers, retailers, consumers and Ghana storage industry is 5% [39]. From the study, %IDK in the PP bags at the end of the 6 months of storage was approximately 67%, whereas that of ZFH and PICS bags were ~3 and 5%, respectively, making grains in the hermetic bags wholesome for consumption compared to that from PP bags.

The results of the study showed that per cent weight loss in the PP bag was approximately 30% at the end of six months of storage, whereas that of the hermetic bag was 0.23%. In a related study by [33], more than 11% grain weight loss was observed when maize was stored in PP bags for six months.

Exposure to mycotoxins is widespread in West Africa [14]. The economic and health importance of mycotoxins needs greater attention because of their ability to contaminate human food and animal feeds, especially cereals [40]. The results of this study showed that the levels of aflatoxin and fumonisin in all the storage bags were below the safe threshold of 15 ppb and 4 ppm, respectively [39]. However, levels of aflatoxin and fumonisin in the PP storage bag increased from 11.56 ppb in the first month to 14.78 ppb in the sixth month. The increase was likely due to high insect infestation, and subsequent increase in IDK and fungal infection. Ref. [41] reported that aflatoxin contamination was comparatively higher in insect-damaged maize than insect-free maize.

The nutritional quality of stored maize is affected by insect pests and rodents because of their feeding activities [42]. Storage pests cause significant qualitative losses, ultimately reducing nutritional quality [43]. This helps explain why significant differences were observed between PP bags and the hermetic bags for all the variables except %ASH. The decrease in nutrient content in the maize stored in the PP bags may be attributed to the insect infestation and damage to the maize. It is reported by [44] that insects that are internal feeders consume parts of seeds which contain substantial amounts of the nutrients, especially crude protein [45]. The distribution of protein in maize has been reported as ~13.2% in bran, 8.6% in endosperm and 34.4% in the germ layer of the kernel. In this study, initial and final proximate analyses were similar for all variables in the PICS and ZFH bags. Maize stored in ZFH and PICS bags had higher nutrient levels than that in PP bags because of the low insect infestation in the hermetic bags. This observation is supported by [46] who reported slower depletion of nutrients in maize stored under hermetic conditions compared to traditional polypropylene bags.

Based on this study, both PICS and ZFH bags protected stored maize by killing insects already in the storage bags. The ZFH and PICS bags maintained the nutrient content of stored maize, and also kept the aflatoxin and fumonisin within safe levels for human and animal use. Therefore, poultry farmers, maize aggregators, and other stakeholders are encouraged to employ hermetic storage bags to store maize to preserve its quality for food and feed.

## Figures and Tables

**Table 1:** Mean (±SE) percent moisture content (%MC) of maize in Polypropylene (PP), Purdue Improved Crop Storage (PICS), and ZeroFly^®^ Hermetic (ZFH) bags sampled during the months of October 2020–March 2021.

Month	PP	PICS	ZFH
0	12.47 ± 0.03 ^e^	12.62 ± 0.87 ^b^	12.48 ± 0.06
1	13.07 ± 0.14 ^dA^	12.63 ± 0.04 ^bB^	12.55 ± 0.03 ^B^
2	13.44 ± 0.11 ^cA^	12.78 ± 0.01 ^ab^	12.49 ± 0.05 ^C^
3	14.03 ± 0.25 ^bA^	12.61 ± 0.10 ^bB^	12.48 ± 0.04 ^B^
4	13.68 ± 0.06 ^bcA^	12.25 ± 0.12 ^cB^	12.36 ± 0.05 ^B^
5	14.23 ± 0.18 ^bA^	12.32 ± 0.13 ^bcB^	12.42 ± 0.04 ^B^
6	15.51 ± 0.19 ^aA^	12.68 ± 0.05 ^bB^	12.41 ± 0.03 ^B^

Significant differences in %MC levels within type of storage bag for the 6 months are denoted by different lower-case letters and differences for storage bags within each month are denoted by different upper-case letters. Where there are no lower-case or upper-case letters, there are no significant differences. (*p* < 0.05, SAS; SLICE option in an LSMEANS statement).

**Table 2:** Mean (±SE) number of insects in maize in Polypropylene (PP), Purdue Improved Crop Storage (PICS), and ZeroFly^®^ Hermetic (ZFH) bags sampled during the months of October 2020–March 2021.

Month	PP	PICS	ZFH
0	0.83 ± 0.17 ^eA^	1.33 ± 0.44 ^cA^	1.17 ± 0.33 ^bA^
1	44.50 ± 7.75 ^dA^	6.33 ± 0.44 ^abB^	9.00 ±1.15 ^aB^
2	101.33 ±22.04 ^cA^	13.67 ± 4.00 ^aB^	12.17 ±4.60 ^aB^
3	147.50 ± 16.23 ^bA^	8.50 ± 1.61 ^abB^	8.33 ± 1.64 ^aB^
4	284.83 ± 25.58 ^aA^	5.17 ± 0.88 ^bcB^	4.83 ± 0.73 ^abB^
5	266.67 ± 0.83 ^aA^	5.50 ± 0.00 ^bB^	4.33 ± 0.44 ^abB^
6	161.00 ± 4.25 ^bA^	7.00 ± 0.29 ^abB^	4.50 ± 0.76 ^abB^

Significant differences in the number of insects in 500 g samples taken from type of storage bag for the 6 months are denoted by different lower-case letters and differences for storage bags within each month are denoted by different upper-case letters. Where there are no lower-case or upper-case letters, there are no significant differences. (*p* < 0.05, SAS; SLICE option in an LSMEANS statement).

**Table 3:** Mean percent (±SE) of insect-damaged kernels (%IDK) of maize in Polypropylene (PP), Purdue Improved Crop Storage (PICS), and ZeroFly^®^ Hermetic (ZFH) bags sampled during the months of October 2020–March 2021.

Month	PP	PICS	ZFH
0	0.83 ± 0.83 ^dA^	1.00 ± 0.29 ^cA^	0.50 ± 0.29 ^cA^
1	24.67 ± 2.89 ^cA^	6.33 ± 0.83 ^bB^	9.50 ± 1.53 ^aB^
2	41.67 ± 1.59 ^bA^	12.00 ± 2.29 ^aB^	8.67 ± 2.19 ^aB^
3	35.33 ± 5.18 ^bA^	6.00 ± 0.00 ^bB^	7.17 ± 1.09 ^aB^
4	72.00 ± 0.87 ^aA^	6.67 ± 2.05 ^bB^	5.83 ± 1.30 ^aB^
5	76.67 ± 0.44 ^aA^	5.67 ± 0.17 ^bB^	3.67 ± 0.17 ^bB^
6	67.33 ± 2.46 ^aA^	5.17 ± 0.44 ^bB^	2.83 ± 0.33 ^bB^

Significant differences in the number of insect-damaged kernels (IDK) in 500 g samples taken from each type of storage bag for the 6 months are denoted by different lower-case letters and differences for storage bags within each month are denoted by different upper-case letters. (*p* < 0.05, SAS; SLICE option in an LSMEANS statement).

**Table 4:** Mean (±SE) weight loss (%) of maize from Polypropylene (PP), Purdue Improved Crop Storage (PICS), and ZeroFly^®^ Hermetic (ZFH) bags sampled during the months of October 2020–March 2021.

Month	PP	PICS	ZFH
0	0.08 ± 0.08 ^dA^	0.00 ± 0.00 ^aA^	0.00 ± 0.00 ^aA^
1	3.66 ± 1.15 ^cdA^	1.05 ± 0.42 ^aA^	1.15 ± 0.58 ^aA^
2	7.72 ± 2.73 ^bcA^	1.70 ± 0.35 ^aB^	2.76 ± 0.70 ^aB^
3	8.24 ± 4.25 ^bcA^	0.85 ± 0.36 ^aB^	1.16 ± 0.18 ^aB^
4	10.45 ± 1.54 ^bA^	0.46 ± 0.36 ^aB^	0.42 ± 0.42 ^aB^
5	9.41 ± 1.19 ^bA^	3.09 ± 0.36 ^aB^	1.06 ± 0.23 ^aB^
6	29.7 ± 7.07 ^aA^	0.18 ± 0.12 ^aB^	0.23 ± 0.12 ^aB^

Significant differences in weight loss (%) within type of storage bag for the 6 months are denoted by different lower-case letters and differences for storage bags within each month are denoted by different upper-case letters. (*p* < 0.05, SAS; SLICE option in an LSMEANS statement).

**Table 5:** Mean (±SE) levels of aflatoxin (ppb) of maize from Polypropylene (PP), Purdue Improved Crop Storage (PICS), and ZeroFly^®^ Hermetic (ZFH) bags sampled during the months of October 2020–March 2021.

Month	PP	PICS	ZFH
0	9.83 ± 0.54 ^d^	9.68 ± 0.44 ^b^	9.94 ± 0.39
1	11.56 ± 0.21 ^cA^	9.83 ± 0.31 ^abB^	10.36 ± 0.17 ^B^
2	11.89 ± 0.67 ^cA^	10.13 ± 0.15 ^abB^	10.38 ± 0.30 ^B^
3	12.98 ± 0.40 ^bA^	9.81 ± 0.29 ^abB^	10.28 ± 0.39 ^B^
4	13.21 ± 0.19 ^bA^	10.52 ± 0.14 ^abB^	10.31 ± 0.18 ^B^
5	14.10 ± 0.03 ^aA^	9.90 ± 0.16 ^abB^	10.25 ± 0.27 ^B^
6	14.78 ± 0.12 ^aA^	10.62 ± 0.47 ^aB^	10.39 ± 0.18 ^B^

Significant differences in aflatoxin levels within type of storage bag for the 6 months are denoted by different lower-case letters and differences for storage bags within each month are denoted by different upper-case letters. Where there are no lower-case or upper-case letters, there are no significant differences. (*p* < 0.05, SAS; SLICE option in an LSMEANS statement).

**Table 6:** Mean (±SE) levels of fumonisin (ppm) in maize from Polypropylene (PP), Purdue Improved Crop Storage (PICS), and ZeroFly^®^ Hermetic (ZFH) bags sampled during the months of October 2020–March 2021.

Month	PP	PICS	ZFH
0	0.28 ± 0.18 ^d^	0.27 ± 0.01	0.28 ± 0.02
1	0.44 ± 0.01 ^cA^	0.26 ± 0.01 ^B^	0.27 ± 0.01 ^B^
2	0.48 ± 0.03 ^cA^	0.36 ± 0.03 ^B^	0.28 ± 0.02 ^B^
3	0.63 ± 0.03 ^bA^	0.32 ± 0.04 ^B^	0.26 ± 0.01 ^B^
4	0.68 ± 0.00 ^abA^	0.29 ± 0.02 ^B^	0.26 ± 0.01 ^B^
5	0.65 ± 0.12 ^bA^	0.35 ± 0.06 ^B^	0.32 ± 0.02 ^B^
6	0.77 ± 0.03 ^aA^	0.31 ± 0.04 ^B^	0.27 ± 0.01 ^B^

Significant differences in fumonisin levels within type of storage bag for the 6 months are denoted by different lower-case letters and differences for storage bags within each month are denoted by different upper-case letters. Where there are no lower-case or upper-case letters, there are no significant differences. (*p* < 0.05, SAS; SLICE option in an LSMEANS statement).

**Table 7:** Mean (±SE) percentage of crude protein (%CPRO), percentage of crude fat (%CFAT), percentage of carbohydrate (%CHO), percentage of crude fiber (%CFIBER), percentage of ash content (%ASH), and percentage of moisture content (%MC) of maize from Polypropylene (PP), Purdue Improved Crop Storage (PICS), and ZeroFly^®^ Hermetic (ZFH) bags sampled during the months of October 2020 and March 2021.

Variable	Month	PP	PICS	ZFH
%CPRO	0	8.70 ± 0.05 ^a^	8.72 ± 0.03	8.72 ± 0.03
	6	6.31 ± 0.23 ^bB^	8.68 ± 0.05 ^A^	8.75 ± 0.03 ^A^
%CFAT	0	2.37 ± 0.00 ^a^	2.37 ± 0.01	2.38 ± 0.01
	6	1.87 ± 0.09 ^bB^	2.36 ± 0.02 ^A^	2.37 ± 0.01 ^A^
%CHO	0	77.52 ± 0.10 ^a^	77.46 ± 0.01	77.40 ± 0.03
	6	72.82 ± 0.19 ^bB^	77.42 ± 0.02 ^A^	77.42 ± 0.02 ^A^
%FIBER	0	2.37 ± 0.01 ^b^	2.38 ± 0.01	2.37 ± 0.01
	6	3.76 ± 0.61 ^aA^	2.37 ± 0.01 ^B^	2.36 ± 0.01 ^B^
%ASH	0	2.18 ± 0.01	2.18 ± 0.01	2.17 ± 0.00
	6	2.11 ± 0.05	2.17 ± 0.00	2.16 ± 0.02
%MC	0	12.29 ± 0.05 ^b^	12.35 ± 0.05	12.35 ± 0.08
	6	14.69 ± 0.50 ^aA^	12.13 ± 0.08 ^B^	12.19 ± 0.08 ^B^

In the case of each response variable, significant differences within type of storage bag for the two months (start and end months) are denoted by different lower-case letters and differences for storage bags within each month are denoted by different upper-case letters. Where there are no lower-case or upper-case letters, there are no significant differences. (*p* < 0.05, SAS; SLICE option in an LSMEANS statement).

## Data Availability

Data is contained within the article.
